# Metric characterizations for well-posedness of split hemivariational inequalities

**DOI:** 10.1186/s13660-018-1761-4

**Published:** 2018-07-27

**Authors:** Qiao-yuan Shu, Rong Hu, Yi-bin Xiao

**Affiliations:** 1 School of Mathematics and Information Engineering, Chongqing University of Education, Chongqing, P.R. China; 2 School of Mathematical Sciences, University of Electronic Science and Technology of China, Chengdu, P.R. China

**Keywords:** Split hemivariational inequality, Monotone operator, Hemicontinuity, Metric characterization

## Abstract

In this paper, we generalize the concept of well-posedness to a class of split hemivariational inequalities. By imposing very mild assumptions on involved operators, we establish some metric characterizations of the well-posedness for the split hemivariational inequality. The obtained results generalize some related theorems on well-posedness for hemivariational inequalities and variational inequalities in the literature.

## Introduction

The concept of well-posedness, which was firstly introduced by Tykhonov in [1] for a minimization problem and thus was called Tykhonov well-posedness, has been studied widely in recent years for optimization problems, variational inequality problems, hemivariational inequality problems, fixed point problems, saddle point problems, equilibrium problems, and their related problems because of their important applications in physics, mechanics, engineering, economics, management science, etc. (see, for example, [2–13]). Tykhonov well-posedness for an optimization problem is defined by requiring the existence and uniqueness of its solution and the convergence to the unique solution of its approximating sequences. There are a great many kinds of generalizations for the concept of well-posedness, such as Levitin-Polyak well-posedness, parametric well-posedness, and *α*-well-posedness, to optimization problems, variational inequality problems, and their related problems (see, for example, [14–21]).

Due to the close relationship between optimization problems and variational inequality problems, the concept of well-posedness for optimization problems is generalized to variational inequalities and their related problems. The earliest research work of well-posedness for variational inequalities should at least date back to 1980s when Lucchetti and Patrone [22, 23] firstly introduced the concept of well-posedness for a variational inequality and proved some important results. After that, Lignola and Morgan [20], Fang and Hu [24], Huang and Yao [25] have made significant contributions to the study of well-posedness for variational inequalities. As an important generalization of variation inequality, hemivariational inequality has drawn much attention of mathematical researchers due to its abundant applications in mechanics and engineering. With the tools of nonsmooth analysis and nonlinear analysis, many kinds of hemivariational inequalities have been studied since 1980s [7, 26–30]. Also, many kinds of concepts of well-posedness hemivariational inequalities have been studied since Goeleven and Mentagui [31] firstly introduced the concept of well-posedness to a hemivariational inequality in 1995. For more research work on the well-posedness for variational inequalities and hemivariational inequalities, we refer the readers to [14, 20, 32–35].

Split variational inequality, which was introduced by Censor et al. in [36], can be regarded as a generalization of variational inequality and includes as a special case, the split feasibility problem, which is an important model for a wide range of practical problems arising from signal recovery, image processing, and tensity-modulated radiation therapy treatment planning (see, for example, [37–41]). Thus, the concepts of well-posedness and Levitin-Polyak well-posedness for various split variational inequalities were studied by Hu and Fang recently [42]. Obviously, split hemivariational inequality could be regarded as a generalization of split variational inequality. It could arise in a system of hemivariational inequalities for modeling some frictional contact problems in mechanics, where two hemivariational inequalities are linked by a linear constraint. Also, when nonconvex and nonsmooth functionals are involved, the model for the above mentioned practical problems, such as signal recovery and image processing, turns to split hemivariational inequality rather than split variational inequality. However, as far as we know, there are few research works studying well-posedness for split hemivariational inequalities.

Inspired by recent research works on the well-posedness for split variational inequalities and hemivariational inequalities, in this paper, we focus on studying metric characterization of well-posedness for a class of split hemivariational inequalities specified as follows:

Find $(u_{1},u_{2})\in V_{1}\times V_{2}$ such that

SHI $$ \textstyle\begin{cases} u_{2}=Tu_{1}, \\ \langle A_{1}u_{1}-f_{1},v_{1}-u_{1}\rangle_{V_{1}^{*}\times V_{1}}+J_{1}^{\circ}(u_{1};v_{1}-u_{1})\geq0, \quad \forall v_{1}\in V_{1}, \\ \langle A_{2}u_{2}-f_{2},v_{2}-u_{2}\rangle_{V_{2}^{*}\times V_{2}}+J_{2}^{\circ}(u_{2};v_{2}-u_{2})\geq0, \quad \forall v_{2}\in V_{2}, \end{cases} $$

where, for $i=1,2$, $\langle\cdot,\cdot\rangle_{V_{i^{*}}\times V_{i}}$ denotes the duality paring between Banach space $V_{i}$ and its dual space $V_{i}^{*}$, $A_{i}:V_{i}\to V_{i}^{*}$ is an operator from $V_{i}$ to $V_{i}^{*} $, $f_{i}$ is a given point in $V_{i}^{*}$, $J_{i}:V_{i}\to\mathbb{R}$ is a locally Lipschitz functional on $V_{i}$ with $J_{i}^{\circ}(u_{i};v_{i}-u_{i})$ being its generalized directional derivative at $u_{i}$ in direction of $v_{i}-u_{i}$, which will be defined in the next section, and $T:V_{1}\to V_{2}$ is a continuous mapping from $V_{1}$ to $V_{2}$. After defining the concept of well-posedness for the split hemivariational inequality (SHI), we present some metric characterizations for its well-posedness under very mild assumptions.

The remainder of the paper is organized as follows. In Sect. 2, we recall some crucial definitions and results. Under very mild assumptions on involved operators, Sect. 3 presents several results on the metric characterizations of well-posedness for the split hemivariational inequality (SHI). At last, some concluding remarks are provided in Sect. 4.

## Preliminaries

In this section, we recall some useful definitions and key results which will be used to establish the metric characterizations of the split hemivariational inequality (SHI)in the next section and can be found in [7, 29, 43–45].

Let $V_{1}$, $V_{2}$ be two Banach spaces, then the product space **V** of $V_{1}$ and $V_{2}$, i.e., $\mathbf{V}=V_{1}\times V_{2}$, is also a Banach space with the norm $\|\cdot\|_{V_{1}\times V_{2}}$ specified as follows:

$$ \|\mathbf {u}\|_{V_{1}\times V_{2}}=\| u_{1}\| _{V_{1}}+\| u_{2}\|_{V_{2}},\quad \forall\mathbf {u}=(u_{1},u_{2}) \in V_{1}\times V_{2}. $$

The dual paring between the product space **V** and its dual space $\mathbf{V}^{*}$ is

$$\begin{aligned} \bigl\langle \mathbf{u}^{*}, \mathbf{u} \bigr\rangle _{\mathbf{V}^{*}\times \mathbf{V}}=\bigl\langle u_{1}^{*}, u_{1}\bigr\rangle _{V_{1}^{*}\times V_{1}}+\bigl\langle u_{2}^{*}, u_{2}\bigr\rangle _{V_{2}^{*}\times V_{2}}, \quad \forall \mathbf {u}=(u_{1},u_{2})\in\mathbf {V} \mbox{ and } \mathbf{u}^{*}=\bigl(u_{1}^{*},u_{2}^{*}\bigr)\in \mathbf {V}^{*}. \end{aligned}$$

### Definition 2.1

Let V be a Banach space with $V^{\ast}$ being its dual space. Then

1. a sequence $\{{u_{n}}\}\subset V$ is said to be convergent if there exists $u\in V$ such that
  $$ \lim_{n\rightarrow\infty} \|u_{n}-u\|_{V}=0, $$
  which is denoted by $u_{n} \to u$ as $n\to\infty$;
2. a sequence $\{{u_{n}}\}\subset V$ is said to be weakly convergent to a point $u\in V$ if
  $$ \langle f,u_{n}\rangle_{V^{\ast}\times V}\rightarrow\langle f,u \rangle_{V^{\ast}\times V},\quad \forall f\in V^{\ast}, $$
  which is denoted by $u_{n} \rightharpoonup u$ as $n\to\infty$;
3. a sequence $\{u_{n}^{\ast}\}\subset V^{\ast}$ is said to be weakly^∗^ convergent to a point $u^{\ast}\in V^{\ast}$ if
  $$ \bigl\langle u^{\ast}_{n},u\bigr\rangle _{V^{\ast}\times V} \rightarrow\bigl\langle u^{\ast},u\bigr\rangle _{V^{\ast}\times V},\quad \forall u\in V, $$
  which is denoted by $u_{n}^{*}\xrightarrow{w^{\ast}} u^{*}$ as $n\to\infty$.

### Definition 2.2

Let V be a Banach space and $V^{\ast}$ be its dual space. A single-valued operator *A* from *V* to $V^{\ast}$ is said to be

1. monotone if
  $$ \langle Au-Av,u-v\rangle_{V^{\ast}\times V}\geq0,\quad \forall u,v\in V; $$
2. strictly monotone if
  $$ \langle Au-Av,u-v\rangle_{V^{\ast}\times V}>0,\quad \forall u,v\in V \mbox{ and } u \neq v; $$
3. relaxed monotone if there exists a constant $c>0$ such that
  $$ \langle Au-Av,u-v\rangle_{V^{\ast}\times V}\geq-c\|u-v\| _{V}^{2}, \quad \forall u,v\in V; $$
4. strongly monotone if there exists a constant $c>0$ such that
  $$ \langle Au-Av,u-v\rangle_{V^{\ast}\times V}\geq c\|u-v\| _{V}^{2}, \quad \forall u,v\in V. $$

### Definition 2.3

Let *V* be a Banach space and $V^{\ast}$ be its dual space. An operator *T* from *V* to $V^{\ast}$ is said to be

1. continuous if, for any sequence $\{u_{n}\}\subset V$ converging to $u\in V$, $Tu_{n}\to Tu$ in $V^{\ast}$;
2. demicontinuous if, for any sequence $\{u_{n}\}\subset V$ converging to $u\in V$, $Tu_{n}\rightharpoonup Tu$ in $V^{\ast}$;
3. hemicontinuous if, for any $u,v,w\in V$, the function $t\rightarrow\langle T(u+tv),w\rangle_{V^{\ast}\times V}$ is continuous on $[0,1]$;
4. weakly^∗^ continuous (or continuous with respect to weak^∗^ topology for $V^{*}$) if, for any sequence $\{ u_{n}\}\subset V$ converging to $u\in V$, $Tu_{n}\xrightarrow{w^{\ast}} Tu$ in $V^{\ast}$.

### Remark 2.1

In [7, 44], demicontinuity of an operator *T* from *V* to $V^{*}$ is defined by its continuity from *V* to its dual space $V^{*}$ endowed with weak^∗^ topology, which is called here weak^∗^ continuity. In this paper, we define the demicontinuity of an operator *T* from *V* to $V^{*}$ by its continuity from *V* to its dual space $V^{*}$ endowed with weak topology, which is commonly used in most literature works.

### Proposition 2.1

*Let* *V* *be a Banach space with* $V^{\ast}$ *being its dual space and* $T:V\to V^{*}$ *be an operator*. *If* *T* *is continuous*, *then it is weakly*^∗^ *continuous*, *which*, *in turn*, *implies that it is hemicontinuous*. *Moreover*, *if* *T* *is a monotone operator*, *then the notions of weak*^∗^ *continuity and hemicontinuity coincide* [7, 44].

### Proposition 2.2

*Let* *V* *be a Banach space with* $V^{*} $ *being its dual space*, *and* $T:V\to V^{*}$ *is a operator from* *V* *to* $V^{*}$. *Then the following statement holds*:

*If* $\{u_{n}\}\subset V$, $\{Tu_{n}\}\subset V^{*}$, $u_{n}\rightarrow u$ *in* *V* *and* $Tu_{n}\xrightarrow{w^{\ast}} Tu$ *in* $V^{*}$, *then*

$$ \langle Tu_{n},u_{n}\rangle_{V^{*}\times V} \rightarrow \langle Tu,u\rangle _{V^{*}\times V}. $$

### Definition 2.4

Let *V* be a Banach space and $J:V\rightarrow\mathbb{R}$ be a functional on *V*. *J* is said to be Lipschitz continuous on *V* if there exists a constant $L>0$ such that

$$ \bigl\vert J(u_{1})-J(u_{2}) \bigr\vert \leq L \|u_{1}-u_{2}\|_{V},\quad \forall u_{1}, u_{2}\in V. $$

### Definition 2.5

Let V be a Banach space and $J:V\rightarrow\mathbb{R}$ be a functional on *V*. *J* is said to be locally Lipschitz continuous on *V* if, for all $u\in V$, there exist a neighborhood $\mathscr {N}(u)$ and a constant $L_{u}>0$ such that

$$ \bigl\vert J(u_{1})-J(u_{2}) \bigr\vert \leq L_{u}\|u_{1}-u_{2}\|_{V},\quad \forall u_{1}, u_{2}\in \mathscr {N}(u). $$

### Definition 2.6

Let *V* be a Banach space and the generalized directional derivative (in the sense of Clarke) of the locally Lipschitz function $J:V\rightarrow \mathbb{R}$ at a point of $u\in V$ in the direction $v\in V$, denoted by $J^{\circ}(u;v)$ and defined by

$$ J^{\circ}(u;v)=\limsup_{w \to u,\lambda\downarrow0}\frac{J(w+\lambda v)-J(w)}{\lambda}. $$

### Definition 2.7

Let *V* be a Banach space and $J:V\rightarrow\mathbb{R}$ be a locally Lipschitz function. Then the generalized gradient in the sense of Clarke of *J* at $v\in V$, denoted by $\partial J(u)$, is the subset of its dual space $V^{\ast}$ defined by

$$ \partial J(u)=\bigl\{ \zeta\in V^{\ast}:J^{\circ}(u;v)\geq\langle \zeta ,v\rangle_{V^{\ast}\times V}, \forall v\in V \bigr\} . $$

### Definition 2.8

Let *A* be a nonempty subset of Banach space *V*. The measure of noncompactness *μ* of the set *A* is defined by

$$ \mu(A)=\inf\Biggl\{ \epsilon>0:A\subset\bigcup_{i=1}^{n}A_{i}, \operatorname{diam} A_{i}< \epsilon,i=1,2,3,\ldots,n\Biggr\} , $$

where diam denotes the diameter of the subset $A_{i}$.

### Definition 2.9

Let *A*, *B* be two nonempty subsets of Banach space *V*. The Hausdorff metric $\mathscr {H}(\cdot,\cdot)$ between *A* and *B* is defined by

$$ \mathscr {H}(A,B)=\max\bigl\{ e(A,B),e(B,A)\bigr\} , $$

where $e(A,B)=\sup_{a\in A}d(a,B)$ with $d(a,B)=\inf_{b\in B}\|a-b\|_{V}$.

### Proposition 2.3

*Let* *V* *be a Banach space and* $V^{*} $ *be its dual space*, $J:V\to\mathbb {R}$ *be a locally Lipschitz functional on* *V*, *and* $u,v\in V$ *be two given elements*. *Then*

1. *the function* $v\to J^{\circ}(u,v)$ *is finite*, *positively homogeneous*, *and subadditive*, *i*.*e*.,
  $$\begin{aligned} J^{\circ}(x;\lambda v)=\lambda J^{\circ}(x;v),\quad \forall \lambda \geq0, \end{aligned}$$
  *and*
  $$\begin{aligned} J^{\circ}(x; v_{1}+v_{2})=J^{\circ}(x;v_{1})+ J^{\circ}(x;v_{2}), \quad \forall v_{1},v_{2} \in V; \end{aligned}$$
2. $J^{\circ}(u,v)$ *is upper semicontinuous on* $V\times V$ *as a function of* $(u,v)$, *i*.*e*., *for all* $u,v\in V$, ${u_{n}}\subset V$, ${v_{n}}\subset V$ *such that* $u_{n}\to u$, $v_{n}\to v$ *in* *V*, *we have*
  $$ \limsup_{n\rightarrow\infty} J^{\circ}(u_{n};v_{n}) \leq J^{\circ}(u;v). $$

## Well-posedness and metric characterizations

In this section, we aim to extend the well-posedness to the split hemivariational inequality (SHI). We first give the definition of well-posedness for the split hemivariational inequality (SHI), and then we prove its metric characterizations for the well-posedness by using two useful sets defined.

### Definition 3.1

A sequence $\{(u_{1}^{n},u_{2}^{n})\}\subset V_{1}\times V_{2}$ is called an approximating sequence for the split hemivariational inequality (SHI) if there exists $0<\epsilon_{n}\to0$ such that

$$ \textstyle\begin{cases} \| u_{2}^{n}-Tu_{1}^{n} \|_{V_{2}}\leq\epsilon_{n}, \\ \langle A_{1}u_{1}^{n}-f_{1},v_{1}-u_{1}^{n}\rangle_{V_{1}^{*}\times V_{1}}+J_{1}^{\circ}(u_{1}^{n};v_{1}-u_{1}^{n})\geq-\epsilon_{n}\| v_{1}-u_{1}^{n}\|_{V_{1}},\quad \forall v_{1}\in V_{1}, \\ \langle A_{2}u_{2}^{n}-f_{2},v_{2}-u_{2}^{n}\rangle_{V_{2}^{*}\times V_{2}}+J_{2}^{\circ}(u_{2}^{n};v_{2}-u_{2}^{n})\geq-\epsilon_{n}\| v_{2}-u_{2}^{n}\|_{V_{2}},\quad \forall v_{2}\in V_{2}. \end{cases} $$

### Definition 3.2

The split hemivariational inequality (SHI) is said to be strongly (resp., weakly) well-posed if it has a unique solution and every approximating sequence for the split hemivariational inequality (SHI) converges strongly (resp., weakly) to the unique solution.

### Definition 3.3

The split hemivariational inequality (SHI) is said to be well-posed in generalized sense (or generalized well-posed) if its solution set is nonempty and, for every approximating sequence, there always exists a subsequence converging to some point of its solution set.

In order to establish the metric characterizations for well-posedness of the split hemivariational inequality (SHI), we first define two sets on $V_{1}\times V_{2}$ as follows: for $\varepsilon>0$,

$$\begin{aligned}& \Omega(\epsilon)=\left \{ (u_{1},u_{2})\in V_{1}\times V_{2} :\vphantom{\textstyle\begin{array}{l} \|u_{2}-Tu_{1}\|_{V_{2}}\leq\epsilon, \mbox{and } \forall v_{1}\in V_{1},v_{2}\in V_{2}, \\ \langle A_{1}u_{1}-f_{1},v_{1}-u_{1}\rangle_{V_{1}^{\ast}\times V_{1}}+ J_{1}^{\circ}(u_{1};v_{1}-u_{1})\geq-\varepsilon\|v_{1}-u_{1}\| _{V_{1}}, \\ \langle A_{2}u_{2}-f_{2},v_{2}-u_{2}\rangle_{V_{2}^{\ast}\times V_{2}}+ J_{2}^{\circ}(u_{2};v_{2}-u_{2})\geq-\varepsilon\|v_{2}-u_{2}\|_{V_{2}} \end{array}\displaystyle }\right. \\& \hphantom{\Omega(\epsilon)={}}{} \left.\textstyle\begin{array}{l} \|u_{2}-Tu_{1}\|_{V_{2}}\leq\epsilon, \mbox{and } \forall v_{1}\in V_{1},v_{2}\in V_{2}, \\ \langle A_{1}u_{1}-f_{1},v_{1}-u_{1}\rangle_{V_{1}^{\ast}\times V_{1}}+ J_{1}^{\circ}(u_{1};v_{1}-u_{1})\geq-\varepsilon\|v_{1}-u_{1}\| _{V_{1}}, \\ \langle A_{2}u_{2}-f_{2},v_{2}-u_{2}\rangle_{V_{2}^{\ast}\times V_{2}}+ J_{2}^{\circ}(u_{2};v_{2}-u_{2})\geq-\varepsilon\|v_{2}-u_{2}\|_{V_{2}} \end{array}\displaystyle \right \}, \\& \Psi(\epsilon)=\left \{ (u_{1},u_{2})\in V_{1}\times V_{2} :\vphantom{\textstyle\begin{array}{l} \|u_{2}-Tu_{1}\|_{V_{2}}\leq\epsilon, \mbox{and } \forall v_{1}\in V_{1},v_{2}\in V_{2}, \\ \langle A_{1}v_{1}-f_{1},v_{1}-u_{1}\rangle_{V_{1}^{\ast}\times V_{1}}+ J_{1}^{\circ}(u_{1};v_{1}-u_{1})\geq-\varepsilon\|v_{1}-u_{1}\| _{V_{1}}, \\ \langle A_{2}v_{2}-f_{2},v_{2}-u_{2}\rangle_{V_{2}^{\ast}\times V_{2}}+ J_{2}^{\circ}(u_{2};v_{2}-u_{2})\geq-\varepsilon\|v_{2}-u_{2}\|_{V_{2}} \end{array}\displaystyle }\right. \\& \hphantom{\Psi(\epsilon)={}}\left. \textstyle\begin{array}{l} \|u_{2}-Tu_{1}\|_{V_{2}}\leq\epsilon, \mbox{and } \forall v_{1}\in V_{1},v_{2}\in V_{2}, \\ \langle A_{1}v_{1}-f_{1},v_{1}-u_{1}\rangle_{V_{1}^{\ast}\times V_{1}}+ J_{1}^{\circ}(u_{1};v_{1}-u_{1})\geq-\varepsilon\|v_{1}-u_{1}\| _{V_{1}}, \\ \langle A_{2}v_{2}-f_{2},v_{2}-u_{2}\rangle_{V_{2}^{\ast}\times V_{2}}+ J_{2}^{\circ}(u_{2};v_{2}-u_{2})\geq-\varepsilon\|v_{2}-u_{2}\|_{V_{2}} \end{array}\displaystyle \right \}. \end{aligned}$$

With the definition of two sets $\Omega(\epsilon)$ and $\Psi (\epsilon)$, we can get the following properties.

### Lemma 3.1

*Let* $V_{1}$, $V_{2}$ *be two Banach spaces with* $V_{1}^{\ast}$, $V_{2}^{\ast}$ *being their dual spaces*, *respectively*. *Suppose that*, *for* $i=1,2$, $A_{i}:V_{i}\rightarrow V_{i}^{\ast}$ *is monotone and hemicontinuous on* $V_{i}$ *and* $J_{i}:V_{i}\rightarrow\mathbb{R}$ *is a locally Lipschitz functional*. *Then* $\Omega(\epsilon)=\Psi(\epsilon)$ *for any* $\epsilon>0$.

### Proof

First, we prove $\Omega(\epsilon)\subset\Psi(\epsilon)$ for any $\epsilon>0$. In fact, let ${\mathbf {u}}=(u_{1},u_{2})\in\Omega(\epsilon)$. By the monotonicity of the operators $A_{1}$ and $A_{2}$, it is easy to show that, for any $v_{1}\in V_{1}$ and $v_{2}\in V_{2}$,

$$ \langle A_{1}v_{1},v_{1}-u_{1} \rangle_{V_{1}^{\ast}\times V_{1}} \geq\langle A_{1}u_{1},v_{1}-u_{1} \rangle_{V_{1}^{\ast}\times V_{1}} $$

and

$$ \langle A_{2}v_{2},v_{2}-u_{2} \rangle_{V_{2}^{\ast}\times V_{2}}\geq\langle A_{2}u_{2},v_{2}-u_{2} \rangle_{V_{2}^{\ast}\times V_{2}}, $$

which imply that

$$\begin{aligned}& \langle A_{1}v_{1}-f_{1},v_{1}-u_{1} \rangle_{V_{1}^{\ast}\times V_{1}}+J_{1}^{\circ }(u_{1};v_{1}-u_{1}) \\& \quad \geq \langle A_{1}u_{1}-f_{1},v_{1}-u_{1} \rangle _{V_{1}^{\ast}\times V_{1}}+J_{1}^{\circ}(u_{1};v_{1}-u_{1}) \\& \quad \geq -\epsilon\|v_{1}-u_{1}\|_{V_{1}} \end{aligned}$$

and

$$\begin{aligned}& \begin{aligned} &\langle A_{2}v_{2}-f_{2},v_{2}-u_{2} \rangle_{V_{2}^{\ast}\times V_{2}}+J_{2}^{\circ }(u_{2};v_{2}-u_{2}) \\ &\quad \geq \langle A_{2}u_{2}-f_{2},v_{2}-u_{2} \rangle _{V_{2}^{\ast}\times V_{2}}+J_{2}^{\circ}(u_{2};v_{2}-u_{2}) \\ &\quad \geq -\epsilon\|v_{2}-u_{2}\|_{V_{2}}. \end{aligned} \end{aligned}$$

This together with the fact that $\|u_{2}-Tu_{1}\|_{V_{2}}\leq\epsilon$ due to ${\mathbf {u}}=(u_{1},u_{2})\in\Omega(\epsilon)$ indicates that $\mathbf {u}\in\Psi(\epsilon)$, and thus $\Omega(\epsilon)\subset\Psi (\epsilon)$.

Now, we turn to prove $\Psi(\epsilon)\subset\Omega(\epsilon)$ for any $\epsilon>0$. Let $\mathbf{u}=(u_{1},u_{2})\in\Psi(\epsilon)$, and then

3.1 $$\begin{aligned} \textstyle\begin{cases} \|u_{2}-Tu_{1}\|_{V_{2}}\leq\epsilon, \quad \mbox{and}\quad \forall v_{1}\in V_{1},v_{2}\in V_{2}, \\ \langle A_{1}v_{1}-f_{1},v_{1}-u_{1}\rangle_{V_{1}^{\ast}\times V_{1}} +J_{1}^{\circ}(u_{1};v_{1}-u_{1})\geq-\epsilon\|v_{1}-u_{1}\|_{V_{1}}, \\ \langle A_{2}v_{2}-f_{2},v_{2}-u_{2}\rangle_{V_{2}^{\ast}\times V_{2}}+J_{2}^{\circ}(u_{2};v_{2}-u_{2})\geq-\epsilon\|v_{2}-u_{2}\|_{V_{2}}. \end{cases}\displaystyle \end{aligned}$$

Let $\mathbf{w}=(w_{1},w_{2})$ be any point in $V_{1}\times V_{2}$ and $t\in (0,1]$. Substituting $v_{1}=u_{1}+t(w_{1}-u_{1})$, $v_{2}=u_{2}+t(w_{2}-u_{2})$ in above inequality (3.1) yields that

3.2 $$\begin{aligned} \textstyle\begin{cases} \|u_{2}-Tu_{1}\|_{V_{2}}\leq\epsilon, \\ \langle A_{1}(u_{1}+t(w_{1}-u_{1}))-f_{1},t(w_{1}-u_{1})\rangle _{V_{1}^{\ast}\times V_{1}} +J_{1}^{\circ}(u_{1};t(w_{1}-u_{1})) \\ \quad \geq-\epsilon\|t(w_{1}- u_{1})\|_{V_{1}}, \\ \langle A_{2}(u_{2}+t(w_{2}-u_{2}))-f_{2},t(w_{2}-u_{2})\rangle _{V_{2}^{\ast}\times V_{2}} +J_{2}^{\circ}(u_{2};t(w_{2}-u_{2})) \\ \quad \geq-\epsilon\|t(w_{2}- u_{2})\|_{V_{2}}. \end{cases}\displaystyle \end{aligned}$$

From Proposition 2.3, the function $J_{i}^{\circ}(u_{i},\cdot)$, $i=1,2$, is positively homogeneous. Letting $t\rightarrow0^{+}$ in the last two inequalities of (3.2), it follows from the hemicontinuity of the operators $A_{1}$ and $A_{2}$ that

3.3 $$\begin{aligned} \textstyle\begin{cases} \|u_{2}-Tu_{1}\|_{V_{2}}\leq\epsilon, \\ \langle A_{1}u_{1}-f_{1},w_{1}-u_{1}\rangle_{V_{1}^{\ast}\times V_{1}} +J_{1}^{\circ}(u_{1};w_{1}-u_{1})\geq-\varepsilon\|w_{1}-u_{1}\|_{V_{1}}, \\ \langle A_{2}u_{2}-f_{2},w_{2}-u_{2}\rangle_{V_{2}^{\ast}\times V_{2}} +J_{2}^{\circ}(u_{2};w_{2}-u_{2})\geq-\varepsilon\|w_{2}-u_{2}\|_{V_{2}}. \end{cases}\displaystyle \end{aligned}$$

By the arbitrariness of $\mathbf {w}=(w_{1},w_{2})\in V_{1}\times V_{2}$, we conclude that $\mathbf {u} \in\Omega(\varepsilon)$, and thus $\Psi(\epsilon)\subset\Omega(\epsilon)$. This completes the proof of Lemma 3.1. □

### Lemma 3.2

*Let* $V_{1}$, $V_{2}$ *be two reflective Banach spaces with* $V_{1}^{\ast}$, $V_{2}^{\ast}$ *being their dual spaces*, *respectively*, *and* $J_{i}:V_{i}\rightarrow\mathbb{R}$, $i=1,2$, *be a locally Lipschitz functional*. *Suppose that* $T:V_{1}\to V_{2}$ *is a continuous operator from* $V_{1}$ *to* $V_{2}$. *Then*, *for any* $\epsilon>0$, $\Psi(\epsilon)$ *is closed in* $V_{1}\times V_{2}$.

### Proof

Assume that $\{\mathbf{u}^{n}=(u_{1}^{n},u_{2}^{n})\} \subset\Psi(\epsilon)$ and $\mathbf{u}^{n}\to\mathbf {u}=(u_{1},u_{2})$ in $V_{1}\times V_{2}$. It follows that

3.4 $$\begin{aligned} \textstyle\begin{cases} \|u_{2}^{n}-Tu_{1}^{n}\|_{V_{2}}\leq\epsilon,\quad \mbox{and} \quad \forall v_{1}\in V_{1},v_{2}\in V_{2}, \\ \langle A_{1}v_{1}-f_{1},v_{1}-u_{1}^{n}\rangle_{V_{1}^{\ast}\times V_{1}} +J_{1}^{\circ}(u_{1}^{n};v_{1}-u_{1}^{n})\geq-\epsilon\|v_{1}-u_{1}^{n}\|_{V_{1}}, \\ \langle A_{2}v_{2}-f_{2},v_{2}-u_{2}^{n}\rangle_{V_{2}^{\ast}\times V_{2}} +J_{2}^{\circ}(u_{2}^{n};v_{2}-u_{2}^{n})\geq-\epsilon\|v_{2}-u_{2}^{n}\|_{V_{2}}. \end{cases}\displaystyle \end{aligned}$$

By Proposition 2.3, $J_{i}^{\circ}(\cdot\, ; \cdot)$, $i=1,2$, is upper continuous on $V_{i}\times V_{i}$. By taking lim sup with $n\to +\infty$ on both sides of the last two inequalities of (3.4), it follows from the fact $u_{i}^{n}\to u_{i}$, $i=1,2$, that

$$\begin{aligned}& \langle A_{1}v_{1}-f_{1},v_{1}-u_{1} \rangle_{V_{1}^{\ast}\times V_{1}}+J_{1}^{\circ }(u_{1};v_{1}-u_{1}) \\& \quad \geq \limsup_{n\rightarrow\infty}\bigl\{ \bigl\langle A_{1}v_{1}-f_{1},v_{1}-u_{1}^{n} \bigr\rangle _{V_{1}^{\ast}\times V_{1}}+ J_{1}^{\circ}\bigl(u_{1}^{n};v_{1}-u_{1}^{n} \bigr)\bigr\} \\& \quad \geq -\epsilon\|v_{1}-u_{1}\|_{V_{1}},\quad \forall v_{1}\in V_{1}, \end{aligned}$$

and

$$\begin{aligned}& \langle A_{2}v_{2}-f_{2},v_{2}-u_{2} \rangle_{V_{2}^{\ast}\times V_{2}}+J_{2}^{\circ }(u_{2};v_{2}-u_{2}) \\& \quad \geq \limsup_{n\rightarrow\infty}\bigl\{ \bigl\langle A_{2}v_{2}-f_{2},v_{2}-u_{2}^{n} \bigr\rangle _{V_{2}^{\ast}\times V_{2}}+ J_{2}^{\circ}\bigl(u_{2}^{n};v_{2}-u_{2}^{n} \bigr)\bigr\} \\& \quad \geq -\epsilon\|v_{2}-u_{2}\|_{V_{2}}, \quad \forall v_{2}\in V_{2}. \end{aligned}$$

To complete the proof, we only need to prove $\|u_{2}-Tu_{1}\|_{V_{2}}\leq \epsilon$. Since, for any $n\in\mathbb{N}$, ${\mathbf {u}}^{n}=(u_{1}^{n},u_{2}^{n})\in\Psi(\epsilon)$, it follows that $\| u_{2}^{n}-Tu_{1}^{n} \|_{V_{2}} \leq\epsilon$, which together with the continuity of the functional $\|\cdot\|_{V_{2}}:V_{2}\to\mathbb{R}$ and the operator *T* implies that

$$\begin{aligned} \|u_{2}-Tu_{1}\|_{V_{2}}\leq\epsilon. \end{aligned}$$

Thus ${\mathbf {u}}=(u_{1},u_{2})\in\Psi(\epsilon)$, which implies that $\Psi (\epsilon)$ is closed on $V_{1}\times V_{2}$. This completes the proof of Lemma 3.2. □

With Lemmas 3.1 and 3.2, it is easy to get the following corollary on the closedness of $\Omega(\epsilon)$ for any $\epsilon>0$, which is crucial to the metric characterizations for well-posedness of the split hemivariational inequality (SHI).

### Corollary 3.1

*Let* $V_{1}$, $V_{2}$ *be two Banach spaces with* $V_{1}^{\ast}$, $V_{2}^{\ast}$ *being their dual spaces*, *respectively*. *Suppose that*, *for* $i=1,2$, $A_{i}:V_{i}\rightarrow V_{i}^{\ast}$ *is monotone and hemicontinuous on* $V_{i}$, $J_{i}:V_{i}\rightarrow\mathbb{R}$ *is a locally Lipschitz functional*, *and* $T:V_{1}\to V_{2}$ *is a continuous operator from* $V_{1}$ *to* $V_{2}$. *Then* $\Omega(\epsilon)$ *is closed for any* $\epsilon>0$.

### Remark 3.1

Similar to the idea in many research works on well-posedness for variational inequalities and hemivariational inequalities [17, 25, 46, 47], the set $\Psi (\epsilon)$ is defined to prove the closedness of $\Omega(\epsilon)$ under the condition that, for $i=1,2$, $A_{i}$ is monotone and hemicontinuous on $V_{i}$. Actually, without defining the set $\Psi (\epsilon)$, we could prove directly the property of closedness of $\Omega(\epsilon)$.

### Lemma 3.3

*Let* $V_{1}$, $V_{2}$ *be two Banach spaces with* $V_{1}^{\ast}$, $V_{2}^{\ast}$ *being their dual spaces*, *respectively*, *and* $J_{i}:V_{i}\rightarrow\mathbb{R}$ *be a locally Lipschitz functional for* $i=1,2$. *Suppose that* $T:V_{1}\to V_{2}$ *is a continuous operator from* $V_{1}$ *to* $V_{2}$ *and for* $i=1,2$, $A_{i}:V_{i}\rightarrow V_{i}^{\ast}$ *is monotone and hemicontinuous*. *Then* $\Omega(\epsilon)$ *is closed for any* $\epsilon>0$.

### Proof

Let $\{\mathbf{u}^{n}=(u_{1}^{n},u_{2}^{n})\}\subset \Omega(\epsilon)$ be a sequence converging to $\mathbf {u}=(u_{1},u_{2})$ in $V_{1}\times V_{2}$, which implies that

3.5 $$\begin{aligned} \textstyle\begin{cases} \|u_{2}^{n}-Tu_{1}^{n}\|_{V_{2}}\leq\epsilon, \quad \mbox{and} \quad \forall v_{1}\in V_{1},v_{2}\in V_{2}, \\ \langle A_{1}u_{1}^{n}-f_{1},v_{1}-u_{1}^{n}\rangle_{V_{1}^{\ast}\times V_{1}} +J_{1}^{\circ}(u_{1}^{n};v_{1}-u_{1}^{n})\geq-\epsilon\|v_{1}-u_{1}^{n}\|_{V_{1}}, \\ \langle A_{2}u_{2}^{n}-f_{2},v_{2}-u_{2}^{n}\rangle_{V_{2}^{\ast}\times V_{2}} +J_{2}^{\circ}(u_{2}^{n};v_{2}-u_{2}^{n})\geq-\epsilon\|v_{2}-u_{2}^{n}\|_{V_{2}}. \end{cases}\displaystyle \end{aligned}$$

Since, for $i=1,2$, $A_{i}:V_{i}\rightarrow V_{i}^{\ast}$ is monotone and hemicontinuous, it is weakly^∗^ continuous on $V_{i}$ by Proposition 2.1 and thus $A_{i}u_{i}^{n}\xrightarrow{w^{\ast}} A_{i}u_{i}$ when $n\to \infty$. This together with the convergence of $\{u_{i}^{n}\}$ and Proposition 2.2 implies that

3.6 $$\begin{aligned} \lim_{n\rightarrow\infty}\bigl\langle A_{1}u_{1}^{n}-f_{1},v_{1}-u_{1}^{n} \bigr\rangle _{V_{1}^{\ast}\times V_{1}}=\langle A_{1}u_{1}-f_{1},v_{1}-u_{1} \rangle_{V_{1}^{\ast }\times V_{1}} \end{aligned}$$

and

3.7 $$\begin{aligned} \lim_{n\rightarrow\infty}\bigl\langle A_{2}u_{2}^{n}-f_{2},v_{2}-u_{2}^{n} \bigr\rangle _{V_{2}^{\ast}\times V_{2}}=\langle A_{2}u_{2}-f_{2},v_{2}-u_{2} \rangle_{V_{2}^{\ast }\times V_{2}}. \end{aligned}$$

By Proposition 2.3, $J_{i}^{\circ}(\cdot\, ; \cdot)$, $i=1,2$, is upper continuous on $V_{i}\times V_{i}$. Taking lim sup with $n\to +\infty$ on both sides of the last two inequalities of (3.5), it follows from (3.6) and (3.7) that

3.8 $$\begin{aligned}& \langle A_{1}u_{1}-f_{1},v_{1}-u_{1} \rangle_{V_{1}^{\ast}\times V_{1}}+J_{1}^{\circ }(u_{1};v_{1}-u_{1}) \\& \quad \geq \limsup_{n\rightarrow\infty}\bigl\{ \bigl\langle A_{1}u_{1}^{n}-f_{1},v_{1}-u_{1}^{n} \bigr\rangle _{V_{1}^{\ast}\times V_{1}}+ J_{1}^{\circ}\bigl(u_{1}^{n};v_{1}-u_{1}^{n} \bigr)\bigr\} \\& \quad \geq -\epsilon\|v_{1}-u_{1}\|_{V_{1}}, \quad \forall v_{1}\in V_{1}, \end{aligned}$$

and

3.9 $$\begin{aligned}& \langle A_{2}u_{2}-f_{2},v_{2}-u_{2} \rangle_{V_{2}^{\ast}\times V_{2}}+J_{2}^{\circ }(u_{2};v_{2}-u_{2}) \\& \quad \geq \limsup_{n\rightarrow\infty}\bigl\{ \bigl\langle A_{2}u_{2}^{n}-f_{2},v_{2}-u_{2}^{n} \bigr\rangle _{V_{2}^{\ast}\times V_{2}}+ J_{2}^{\circ}\bigl(u_{2}^{n};v_{2}-u_{2}^{n} \bigr)\bigr\} \\& \quad \geq -\epsilon\|v_{2}-u_{2}\|_{V_{2}},\quad \forall v_{2}\in V_{2}. \end{aligned}$$

Moreover, by similar arguments as in Lemma 3.2, it is easy to show that

3.10 $$\begin{aligned} \|u_{2}-Tu_{1}\|_{V_{2}}\leq\epsilon. \end{aligned}$$

This together with (3.8) and (3.9) indicates that ${\mathbf {u}}=(u_{1},u_{2})\in\Omega(\epsilon)$. Thus $\Omega(\epsilon)$ is closed on $V_{1}\times V_{2}$. This completes the proof of Lemma 3.3. □

Now, with properties of the set $\Omega(\epsilon)$ given above, we are in a position to prove metric characterizations for the split hemivariational inequality (SHI)by using similar methods for studying well-posedness of variational inequalities and hemivariational inequalities in research works [17, 25, 46, 47].

### Theorem 3.1

*Let* $V_{1}$, $V_{2}$ *be two Banach spaces and* $V_{1}^{\ast}$, $V_{2}^{\ast}$ *be their dual spaces*, *respectively*. *Suppose that*, *for* $i=1,2$, $A_{i}:V_{i}\rightarrow V_{i}^{\ast}$ *is an operator on* $V_{i}$ *and* $J_{i}:V_{i}\rightarrow\mathbb{R}$ *is a locally Lipschitz functional*. *Then the split hemivariational inequality* (SHI) *is strongly well*-*posed if and only if its solution set* *S* *is nonempty and* $\operatorname{diam} \Omega(\varepsilon)\rightarrow0$ *as* $\varepsilon\rightarrow0$.

### Proof

“Necessity”: First of all, it is obvious that the solution set of the split hemivariational inequality (SHI) $S\neq\phi$ since it has a unique solution due to its strong well-posedness. Assume that $\operatorname{diam} \Omega(\epsilon)\nrightarrow0$ as $\epsilon\rightarrow0$, then there exist $\delta>0$, $\epsilon_{k}\rightarrow0^{+}$, $\mathbf {u}^{k}=(u_{1}^{k},u_{2}^{k})\in\Omega(\epsilon_{k})$, and $\mathbf {p}^{k}=(p_{1}^{k},p_{2}^{k})\in\Omega(\epsilon_{k})$ such that

3.11 $$ \bigl\Vert \mathbf{u}^{k}-\mathbf{p}^{k} \bigr\Vert _{V_{1}\times V_{2}}= \bigl\Vert \bigl(u_{1}^{k},u_{2}^{k} \bigr)-\bigl(p_{1}^{k},p_{2}^{k}\bigr) \bigr\Vert _{V_{1}\times V_{2}}\geq\delta , \quad \forall k\in\mathbb{N}. $$

Clearly, both $\{(u_{1}^{k},u_{2}^{k})\}$ and $\{(p_{1}^{k},p_{2}^{k})\}$ are approximating sequences for the split hemivariational inequality (SHI) by the fact that $(u_{1}^{k},u_{2}^{k})\in\Omega (\epsilon_{k})$ and $(p_{1}^{k},p_{2}^{k})\in\Omega(\epsilon_{k})$. It follows from the well-posedness of (SHI) that both $\{(u_{1}^{k},u_{2}^{k})\}$ and $\{(p_{1}^{k},p_{2}^{k})\}$ converge to the unique solution of (SHI), which is a contradiction to (3.11). Thus, $\operatorname{diam} \Omega(\varepsilon)\rightarrow0$ as $\varepsilon\rightarrow0$.

“Sufficiency”: Suppose that the solution set *S* of the split hemivariational inequality (SHI) is nonempty and $\operatorname{diam} \Omega(\epsilon)\rightarrow0$ as $\epsilon\rightarrow0$. For any approximating sequence $\{\mathbf{u}^{n}=(u_{1}^{n},u_{2}^{n})\}\subset V_{1}\times V_{2}$ for (SHI), there exists $0<\epsilon_{n}\to0$ such that

3.12 $$\begin{aligned} \textstyle\begin{cases} \| u_{2}^{n}-Tu_{1}^{n} \|_{V_{2}}\leq\epsilon_{n}, \\ \langle A_{1}u_{1}^{n}-f_{1},v_{1}-u_{1}^{n}\rangle_{V_{1}^{*}\times V_{1}}+J_{1}^{\circ}(u_{1}^{n};v_{1}-u_{1}^{n})\geq-\epsilon_{n}\| v_{1}-u_{1}^{n}\|_{V_{1}},\quad \forall v_{1}\in V_{1}, \\ \langle A_{2}u_{2}^{n}-f_{2},v_{2}-u_{2}^{n}\rangle_{V_{2}^{*}\times V_{2}}+J_{2}^{\circ}(u_{2}^{n};v_{2}-u_{2}^{n})\geq-\epsilon_{n}\| v_{2}-u_{2}^{n}\|_{V_{2}},\quad \forall v_{2}\in V_{2}, \end{cases}\displaystyle \end{aligned}$$

which indicates that $(u_{1}^{n},u_{2}^{n})\in\Omega(\varepsilon_{n})$ with $\varepsilon_{n}\rightarrow0$.

Now, we claim that the solution set *S* of the split hemivariational inequality (SHI)is a singleton, i.e., $S=\{\mathbf{u}^{\ast }=(u_{1}^{\ast},u_{2}^{\ast})\}$ and $\mathbf{u}^{n}\to\mathbf{u}^{*}$ as $n\to\infty$, which indicate that the split hemivariational inequality (SHI)is strongly well-posed. For the purpose of getting contradiction, we suppose that there exists another solution $\mathbf {u}^{\prime}=(u_{1}^{\prime},u_{2}^{\prime})\neq\mathbf{u}^{*}$ to the split hemivariational inequality (SHI). It is clear that $\mathbf {u}^{\prime}, \mathbf{u}^{*}\in\Omega(\epsilon)$ for any $\epsilon >0$ and

$$ \bigl\Vert \mathbf{u}^{\ast}-\mathbf{u}^{\prime} \bigr\Vert _{V_{1}\times V_{2}}\leq \operatorname{diam} \Omega(\epsilon)\rightarrow0, \quad \mbox{as } \epsilon\to0, $$

which is a contradiction. Thus, $\mathbf{u}^{*}$ is the unique solution to the split hemivariational inequality (SHI). Moreover, since $\mathbf{u}^{n}$, $\mathbf{u}^{*}\in\Omega(\epsilon_{n})$ for any $n\in \mathbb{N}$, it follows that

$$ \bigl\Vert \mathbf{u}^{n}-\mathbf{u}^{\ast} \bigr\Vert _{V_{1}\times V_{2}}= \bigl\Vert \bigl(u_{1}^{\ast },u_{2}^{\ast} \bigr)-\bigl(u_{1}^{n},u_{2}^{n}\bigr) \bigr\Vert _{V_{1}\times V_{2}}\leq \operatorname{diam} \Omega (\epsilon_{n}) \rightarrow0, \quad \mbox{as } n\to\infty, $$

which implies that $\mathbf{u}^{n}\to\mathbf{u}^{*}$ as $n\to\infty$. This completes the proof of Theorem 3.1. □

### Theorem 3.2

*Let* $V_{1}$, $V_{2}$ *be two Banach spaces with* $V_{1}^{\ast}$, $V_{2}^{\ast}$ *being their dual spaces*, *respectively*, *and* $T:V_{1}\to V_{2}$ *be a continuous operator from* $V_{1}$ *to* $V_{2}$. *Suppose that*, *for* $i=1,2$, $A_{i}:V_{i}\rightarrow V_{i}^{\ast}$ *is monotone and demicontinuous on* $V_{i}$ *and* $J_{i}:V_{i}\rightarrow\mathbb{R}$ *is a locally Lipschitz functional*. *Then the split hemivariational inequality* (SHI) *is strongly well*-*posed if and only if*

3.13 $$\begin{aligned} \Omega(\epsilon)\neq\emptyset, \quad \forall\epsilon>0,\quad \textit {and}\quad \operatorname{diam} \Omega(\epsilon)\rightarrow0 \quad \textit{as } \epsilon\rightarrow0. \end{aligned}$$

### Proof

It is sufficient to prove the sufficiency of Theorem 3.2 since it is easy to get its necessity by Theorem 3.1 due to the fact that $S\subset\Omega(\epsilon)$ for any $\epsilon>0$. First, with condition (3.13), it is easy to show that the split hemivariational inequality (SHI) possesses a unique solution by similar arguments as in the proof of Theorem 3.1. Then, we suppose that $\{\mathbf{u}^{n}=(u_{1}^{n},u_{2}^{n})\}\subset V_{1}\times V_{2}$ is an approximating sequence for the split hemivariational inequality, which indicates that there exists $0<\epsilon_{n}\to0$ such that (3.12) holds and thus $\mathbf {u}^{n}\in\Omega(\epsilon_{n})$. It follows from the condition $\operatorname{diam} \Omega(\epsilon)\rightarrow0 as \epsilon\rightarrow0$ that $\{\mathbf{u}^{n}\}$ is a Cauchy sequence. As a consequence, there exists $\mathbf{u}=(u_{1},u_{2})$ such that $\mathbf{u}^{n}\rightarrow \mathbf{u}$. Now, we show that $\mathbf{u}=(u_{1},u_{2})$ is the unique solution of the split hemivariational inequality (SHI)to get its strong well-posedness. By taking limit on both sides of the first inequality in (3.12), it is easy to get from the continuity of the operation *T* that

3.14 $$ u_{2}=Tu_{1}. $$

Since, for $i=1,2$, the operator $A_{i}:V_{i}\rightarrow V_{i}^{\ast}$ is monotone and the Clarke generalized directional derivative $J_{i}^{\circ }(\cdot\, ; \cdot)$ is upper semicontinuous by Proposition 2.3, taking lim sup on both sides of the last two inequalities in (3.12) yields that

3.15 $$\begin{aligned}& \langle A_{1}v_{1}-f_{1},v_{1}-u_{1} \rangle_{V_{1}^{\ast}\times V_{1}}+J_{1}^{\circ }(u_{1};v_{1}-u_{1}) \\& \quad \geq \limsup_{n\rightarrow\infty}\bigl\{ \bigl\langle A_{1}v_{1}-f_{1},v_{1}-u_{1}^{n} \bigr\rangle _{V_{1}^{\ast}\times V_{1}}+J_{1}^{\circ}\bigl(u_{1}^{n};v_{1}-u_{1}^{n} \bigr)\bigr\} \\& \quad \geq \limsup_{n\rightarrow\infty}\bigl\{ \bigl\langle A_{1}u_{1}^{n}-f_{1},v_{1}-u_{1}^{n} \bigr\rangle _{V_{1}^{\ast}\times V_{1}}+J_{1}^{\circ}\bigl(u_{1}^{n};v_{1}-u_{1}^{n} \bigr)\bigr\} \\& \quad \geq \limsup_{n\rightarrow\infty}-\epsilon_{n} \bigl\Vert v_{1}-u_{1}^{n} \bigr\Vert _{V_{1}} \\& \quad = 0 \end{aligned}$$

and

3.16 $$\begin{aligned}& \langle A_{2}v_{2}-f_{2},v_{2}-u_{2} \rangle_{V_{2}^{\ast}\times V_{2}}+J_{2}^{\circ }(u_{2};v_{2}-u_{2}) \\& \quad \geq \limsup_{n\rightarrow\infty}\bigl\{ \bigl\langle A_{2}v_{2}-f_{2},v_{2}-u_{2}^{n} \bigr\rangle _{V_{2}^{\ast}\times V_{2}}+J_{2}^{\circ}\bigl(u_{2}^{n};v_{2}-u_{2}^{n} \bigr)\bigr\} \\& \quad \geq \limsup_{n\rightarrow\infty}\bigl\{ \bigl\langle A_{2}u_{2}^{n}-f_{2},v_{2}-u_{2}^{n} \bigr\rangle _{V_{2}^{\ast}\times V_{2}}+J_{2}^{\circ}\bigl(u_{2}^{n};v_{2}-u_{2}^{n} \bigr)\bigr\} \\& \quad \geq \limsup_{n\rightarrow\infty}-\epsilon_{n} \bigl\Vert v_{2}-u_{2}^{n} \bigr\Vert _{V_{2}} \\& \quad = 0. \end{aligned}$$

By similar arguments for the proof of $\Psi(\epsilon)\subset\Omega (\epsilon)$ for any $\epsilon>0$ in Lemma 3.1, it can be proved by the hemicontinuity of operators $A_{1}$, $A_{2}$, (3.15), and (3.16) that

$$\begin{aligned} \langle A_{1}u_{1} -f_{1},v_{1}-u_{1} \rangle_{V_{1}^{\ast}\times V_{1}}+J_{1}^{\circ}(u_{1};v_{1}-u_{1}) \geq0 \end{aligned}$$

and

$$\begin{aligned} \langle A_{2}u_{2} -f_{2},v_{2}-u_{2} \rangle_{V_{2}^{\ast}\times V_{2}}+J_{2}^{\circ}(u_{2};v_{2}-u_{2}) \geq0, \end{aligned}$$

which together with (3.14) imply that $\mathbf{u}=(u_{1},u_{2})$ is the unique solution of the split hemivariational inequality (SHI). This completes the proof of Theorem 3.2. □

The following is a concrete example to illustrate the metric characterization of well-posedness for a hemivariational inequality.

### Example 3.1

Let $V_{1}=V_{2}=\mathbb{R}$ and $f_{1}=2$, $f_{2}=1$. For any $u_{1},u_{2}\in \mathbb{R}$, $A_{1}:\mathbb{R}\rightarrow\mathbb{R}$ such that $A_{1}(u_{1})=2u_{1}$, $A_{2}:\mathbb{R}\rightarrow\mathbb{R}$ such that $A_{2}(u_{2})=u_{2}$, $T:\mathbb{R}\rightarrow\mathbb{R}$ such that $T(u_{1})=u_{1}^{2}$, and $J_{1},J_{2}:\mathbb{R}\rightarrow\mathbb{R}$ are defined by

$$ J_{1}(u_{1})= \textstyle\begin{cases} 0 , & \mbox{if } u_{1}< 0,\\ u_{1}^{2}, & \mbox{if } 0\leq u_{1}< 1,\\ 1 , & \mbox{if } u_{1}\geq1, \end{cases}\displaystyle \qquad J_{2}(u_{2})= \textstyle\begin{cases} 0 , & \mbox{if } u_{2}< 0,\\ u_{2}^{2}/2, & \mbox{if } 0\leq u_{2}< 1,\\ 1 , & \mbox{if } u_{2}\geq1. \end{cases} $$

It is obvious that $J_{1}$ and $J_{2}$ are locally Lipschitz and nonconvex functions on $\mathbb{R}$. Thus, the split hemivariational inequality we consider is as follows:

Find $(u_{1},u_{2})\in\mathbb{R}\times\mathbb{R}$ such that

3.17 $$ \textstyle\begin{cases} u_{2}=u_{1}^{2}, \\ (2u_{1}-2)(v_{1}-u_{1})+J_{1}^{\circ}(u_{1};v_{1}-u_{1})\geq0,\quad \forall v_{1}\in \mathbb{R}, \\ (u_{2}-1)(v_{2}-u_{2})+J_{2}^{\circ}(u_{2};v_{2}-u_{2})\geq0,\quad \forall v_{2}\in \mathbb{R}. \end{cases} $$

By some simple calculations, one can easily obtain that the Clarke subgradients for the functions $J_{1}$ and $J_{2}$ are

$$ \partial J_{1}(u_{1})= \textstyle\begin{cases} 2u_{1}, & \mbox{if } 0\leq u_{1}< 1,\\ {[0,2]}, & \mbox{if } u_{1}=1,\\ 0, & \mbox{else}, \end{cases}\displaystyle \qquad \partial J_{2}(u_{2})= \textstyle\begin{cases} u_{2}, & \mbox{if } 0\leq u_{2}< 1,\\ {[0,1]}, & \mbox{if } u_{2}=1,\\ 0, & \mbox{else}. \end{cases} $$

On the one hand, with some further deductions, it is not difficult to check that the split hemivariational inequality (3.17) has a unique solution $\mathbf {u}^{*}=(u_{1}^{*},u_{2}^{*})=(1,1)$. Moreover, for any approximating sequence $\{\mathbf {u}^{n}=(u_{1}^{n},u_{2}^{n})\}$ of the split hemivariational inequality (3.17), it satisfies

3.18 $$ \textstyle\begin{cases} |u_{2}^{n}-u_{1}^{n}{^{2}}|\leq\epsilon_{n}, \quad \mbox{and} \quad \forall v_{1}\in\mathbb{R},v_{2}\in\mathbb{R}, \\ (2u_{1}^{n}-2)(v_{1}-u_{1}^{n})+J_{1}^{\circ}(u_{1}^{n};v_{1}-u_{1}^{n})\geq-\epsilon _{n}|v_{1}-u_{1}^{n}|, \\ (u_{2}^{n}-1)(v_{2}-u_{2}^{n})+J_{2}^{\circ}(u_{2}^{n};v_{2}-u_{2}^{n})\geq-\epsilon_{n} |v_{2}-u_{2}^{n}|, \end{cases} $$

where $0<\epsilon_{n}\rightarrow0$ when $n\rightarrow\infty$. By taking limit of $n\to\infty$ on both sides of the inequalities in (3.18), it is easy to obtain that the approximating sequence $\{\mathbf {u}^{n}\}$ converges strongly to the unique solution $\mathbf {u}^{*}$ of the split hemivariational inequality (3.17), which indicates that the split hemivariational inequality (3.17) is well-posed.

On the other hand, given $\epsilon>0$, $\Omega(\epsilon)$ for the split hemivariational inequality (3.17) is defined by

$$ \Omega(\epsilon)=\left \{ (u_{1},u_{2})\in\mathbb{R} \times\mathbb{R} : \textstyle\begin{array}{l} |u_{2}-u_{1}^{2}|\leq\epsilon, \mbox{and } \forall v_{1}\in \mathbb{R},v_{2}\in\mathbb{R},\\ (2u_{1}-2)(v_{1}-u_{1})+J_{1}^{\circ}(u_{1};v_{1}-u_{1})\geq-\epsilon|v_{1}-u_{1}|,\\ (u_{2}-1)(v_{2}-u_{2})+J_{2}^{\circ}(u_{2};v_{2}-u_{2})\geq-\epsilon|v_{2}-u_{2}| \end{array}\displaystyle \right \}. $$

With some careful calculations, one can specify $\Omega(\epsilon)$ for the split hemivariational inequality (3.17) as follows:

$$ \Omega(\epsilon)= \bigl\{ (u_{1},u_{2})|u_{1} \in[1,1+\epsilon/2],u_{2}\in[1,1+\epsilon],u_{2}\geq u_{1}^{2}-\epsilon \bigr\} . $$

**Figure 1:**
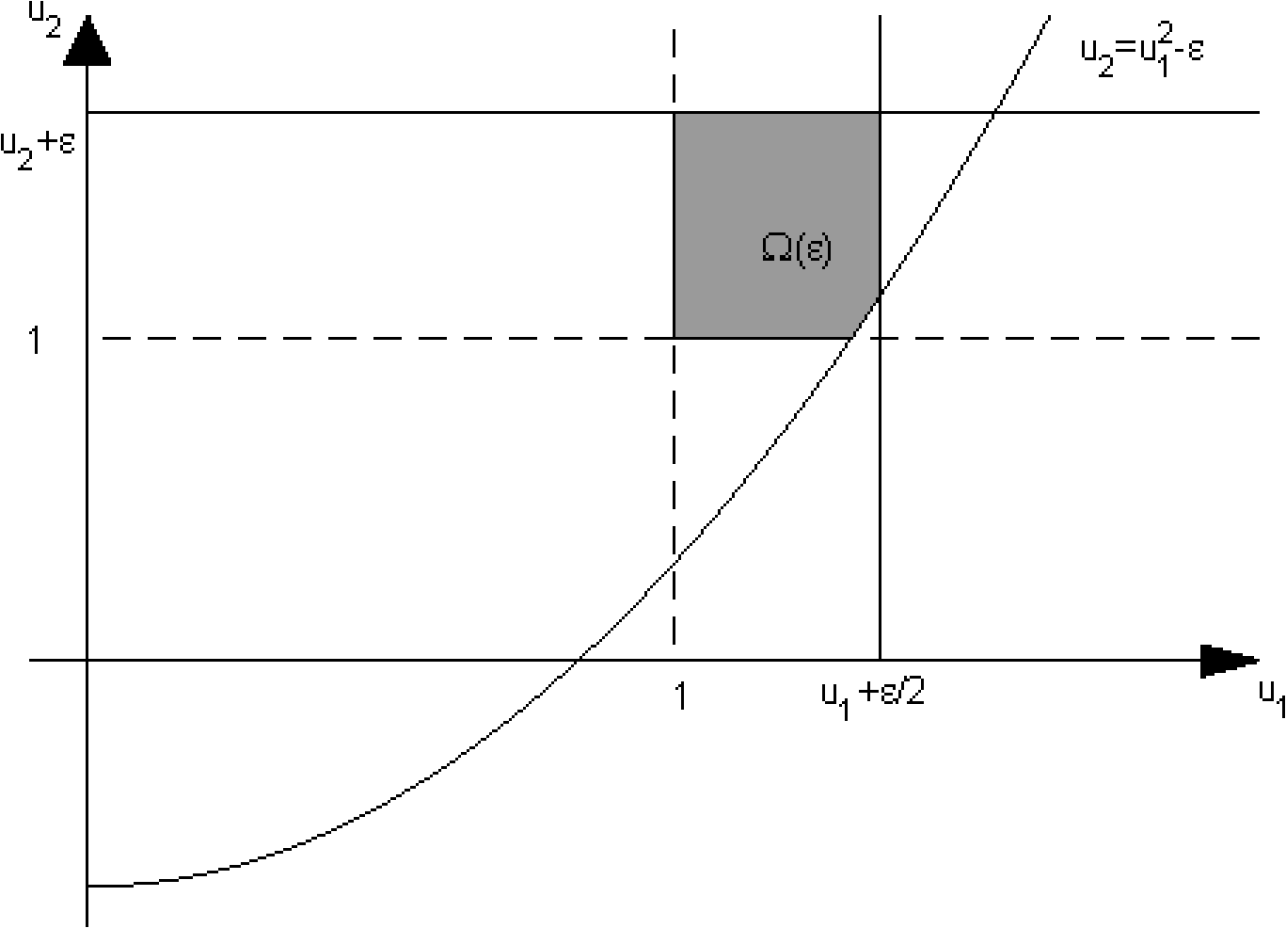
Graph of $\Omega(\epsilon)$

From Fig. 1, the graph of $\Omega(\epsilon)$, it is easy to obtain that

$$\begin{aligned} \operatorname{diam} \Omega(\epsilon)=\sup \bigl\{ \bigl\vert (u_{1},u_{2})-\bigl(u_{1}^{\prime},u_{2}^{\prime}\bigr) \bigr\vert :(u_{1},u_{2}),\bigl(u_{1}^{\prime},u_{2}^{\prime}\bigr)\in\Omega(\epsilon) \bigr\} =\frac{\sqrt{5} \epsilon}{2}. \end{aligned}$$

Obviously, for any $\epsilon>0$, $\Omega(\epsilon)$ for the split hemivariational inequality (3.17) is nonempty and $\operatorname {diam} \Omega(\epsilon)\to0$ when $\epsilon\to0$.

### Theorem 3.3

*Let* $V_{1}$, $V_{2}$ *be two Banach spaces and* $V_{1}^{\ast}$, $V_{2}^{\ast}$ *be their dual spaces*, *respectively*. *Suppose that*, *for* $i=1,2$, $A_{i}:V_{i}\rightarrow V_{i}^{\ast}$ *is an operator on* $V_{i}$ *and* $J_{i}:V_{i}\rightarrow\mathbb{R}$ *is a locally Lipschitz functional*. *Then the split hemivariational inequality* (SHI) *is generalized well*-*posed if and only if its solution set* *S* *is nonempty compact and* $\mathscr {H}(\Omega (\epsilon),S)\rightarrow0$ *as* $\varepsilon\rightarrow0$.

### Proof

First, suppose that the split hemivariational inequality (SHI) is generalized well-posed. This implies, by the definition of generalized well-posedness for (SHI) and the definition of $\Omega(\epsilon)$, that $\phi\neq S\subset \Omega(\epsilon)$ for all $\epsilon>0$. We claim that the solution set *S* of (SHI) is compact. In fact, let $\{\mathbf {u}^{n}=(u_{1}^{n},u_{2}^{n})\}$ be a sequence in *S*, which indicates that $\{\mathbf{u}^{n}\}$ is an approximating sequence for the split hemivariational inequality. By the generalized well-posedness of (SHI), there exists a subsequence of $\{\mathbf{u}^{n}\}$ converging to some element of *S*, which implies that *S* is compact. Now, we prove $\mathscr {H}(\Omega(\epsilon),S)\rightarrow0$ as $\varepsilon \rightarrow0$. If not, there exist $\tau>0$, $\varepsilon_{n}>0$ with $\varepsilon_{n}\to0$, and $\mathbf{u}^{n}=(u_{1}^{n},u_{2}^{n})\in\Omega (\varepsilon_{n})$ such that

3.19 $$\begin{aligned} \mathbf{u}^{n}\nsubseteq S+B(0,\tau),\quad \forall n\in \mathbb{N}. \end{aligned}$$

By the fact that $\mathbf{u}^{n}\in\Omega(\varepsilon_{n})$ for $n\in \mathbb{N}$, $\{\mathbf{u}^{n}\}$ is an approximating sequence for the split hemivariational inequality (SHI), which implies by the generalized well-posedness of (SHI) that there exists a subsequence of $\{\mathbf{u}^{n}\}$ converging to some element of *S*, a contradiction to (3.19). Therefore, $\mathscr {H}(\Omega(\epsilon),S)\rightarrow0$ as $\varepsilon\rightarrow0$.

Conversely, we prove the sufficiency. Assume that *S* is nonempty compact and $\mathscr {H}(\Omega(\epsilon), S)\rightarrow0$ as $\varepsilon\rightarrow0$. For any approximating sequence $\{\mathbf {u}^{n}=(u_{1}^{n},u_{2}^{n})\}$ for the split hemivariational inequality (SHI), there exists $0<\epsilon_{n}\to0$ such that $\{ \mathbf{u}^{n}\}\in\Omega(\varepsilon_{n})$. By virtue of $S\subset \Omega(\epsilon_{n})$ for any $n\in\mathbb{N}$, it is obvious that

$$ d\bigl(\mathbf{u}^{n},S\bigr)\leq e\bigl(\Omega(\varepsilon_{n}),S \bigr)= \max\bigl\{ e\bigl(\Omega(\epsilon),S\bigr),e\bigl(S,\Omega(\epsilon)\bigr) \bigr\} =\mathscr {H}\bigl(\Omega(\epsilon),S\bigr)\rightarrow0. $$

Since *S* is compact, it follows that there exists a sequence $\{ \mathbf{w}^{n}=(w_{2}^{n},w_{1}^{n})\}\subset S$ such that

$$ \bigl\Vert \mathbf{u}^{n}-\mathbf{w}^{n} \bigr\Vert _{V_{1}\times V_{2}}=d\bigl(\mathbf {u}^{n},S\bigr)\rightarrow0. $$

Again by the compactness of the solution set *S* and $\{\mathbf {w}^{n}\}\subset S$, there exists a sequence $\{\mathbf{w}^{n_{k}}\}$ converging to some point $\mathbf{w}^{\prime}\in S$. Thus

$$ \bigl\Vert \mathbf{u}^{n_{k}}-\mathbf{w}^{\prime} \bigr\Vert _{V_{1}\times V_{2}}\leq \bigl\Vert \mathbf{u}^{n_{k}}-\mathbf{w}^{n_{k}} \bigr\Vert _{V_{1}\times V_{2}} + \bigl\Vert \mathbf{w}^{n_{k}}- \mathbf{w}^{\prime} \bigr\Vert _{V_{1}\times V_{2}}\to 0, \quad \mbox{as } k\to \infty, $$

which implies that the split hemivariational inequality (SHI)is generalized well-posed since the solution set *S* for the split hemivariational inequality (SHI)is nonempty. This completes the proof of Theorem 3.3. □

### Theorem 3.4

*Let* $V_{1}$, $V_{2}$ *be two Banach spaces with* $V_{1}^{\ast}$, $V_{2}^{\ast}$ *being their dual spaces*, *respectively*, *and* $T:V_{1}\to V_{2}$ *be a continuous operator from* $V_{1}$ *to* $V_{2}$. *Suppose that*, *for* $i=1,2$, $A_{i}:V_{i}\rightarrow V_{i}^{\ast}$ *is monotone and demicontinuous on* $V_{i}$ *and* $J_{i}:V_{i}\rightarrow\mathbb{R}$ *is a locally Lipschitz functional*. *Then the split hemivariational inequality* (SHI) *is generalized well*-*posed if and only if*

3.20 $$\begin{aligned} \Omega(\epsilon)\neq\emptyset, \quad \forall\epsilon>0, \quad \textit {and} \quad \mu\bigl(\Omega(\epsilon)\bigr)\rightarrow0 \quad \textit{as } \epsilon\rightarrow0. \end{aligned}$$

### Proof

Necessity: With the generalized well-posedness for the split hemivariational inequality (SHI), it is easy to get from Theorem 3.3 that its solution set *S* is nonempty compact and

3.21 $$\begin{aligned} \mathscr {H}\bigl(\Omega(\epsilon),S\bigr)\rightarrow0 \quad \mbox{as } \varepsilon \rightarrow0. \end{aligned}$$

Obviously, $\emptyset\neq S\subset\Omega(\epsilon)$ for any $\epsilon>0$ and, with the compactness of the solution set *S*, (3.21) implies that

$$ \mu\bigl(\Omega(\epsilon)\bigr)\leq2\mathscr {H}\bigl(\Omega(\epsilon),S\bigr)+ \mu (S)=2\mathscr {H}\bigl(\Omega(\epsilon),S\bigr)\to0, \quad \mbox{as } \epsilon \to0. $$

Sufficiency: Conversely, assume that condition (3.20) holds. Note that $S=\bigcap_{\epsilon>0}\Omega(\epsilon)$ due to the closedness of $\Omega(\epsilon)$ for any $\epsilon>0$ by Corollary 3.1. Since $\mu(\Omega(\epsilon))\rightarrow0$ as $\epsilon \rightarrow0$, it follows from the theorem on p. 412 of [45] that *S* is nonempty compact and

$$ e\bigl(\Omega(\epsilon),S\bigr)=\mathscr {H}\bigl(\Omega(\epsilon),S\bigr) \rightarrow 0, \quad \mbox{as } \epsilon\rightarrow0, $$

which implies by Theorem 3.3 that the split hemivariational inequality (SHI) is generalized well-posed. This completes the proof of Theorem 3.4. □

## Concluding remarks

In this paper, we generalize the concept of well-posedness to a split hemivariational inequality (SHI), which is a generalization of classic variational inequality and hemivariational inequality. After defining well-posedness for the split hemivariational inequality (SHI) with its approximating sequences, we establish some metric characterizations using very mild assumptions on operators involved. The obtained results generalize some theorems on well-posedness for hemivariational inequalities and variational inequalities in the literature.

Similar to many research papers on well-posedness for variational inequalities and hemivariational inequalities, in addition to the metric characterizations for well-posedness, it is important and interesting to study the relationships between the well-posedness and its solvability for the split hemivariational inequalities (SHI).
